# Knockout of Density-Enhanced Phosphatase-1 Impairs Cerebrovascular Reserve Capacity in an Arteriogenesis Model in Mice

**DOI:** 10.1155/2013/802149

**Published:** 2013-08-20

**Authors:** Daniel Hackbusch, André Dülsner, Nora Gatzke, Janine Krüger, Philipp Hillmeister, Stephanie Nagorka, Florian Blaschke, Zully Ritter, Christa Thöne-Reineke, Frank-D. Böhmer, Ivo Buschmann, Kai Kappert

**Affiliations:** ^1^Center for Cardiovascular Research (CCR), Institute of Laboratory Medicine, Clinical Chemistry and Pathobiochemistry, Charité-University Medicine Berlin, Hessische Straße 3-4, 10115 Berlin, Germany; ^2^Department of Internal Medicine, Cardiology and Angiology, Richard Thoma Laboratories for Arteriogenesis, Center for Cardiovascular Research, Charité-University Medicine Berlin, Hessische Straße 3-4, 10115 Berlin, Germany; ^3^Department of Cardiology, Charité-University Medicine Berlin, Augustenburger Platz 1, 13353 Berlin, Germany; ^4^Clinic for Radiology and Nuclear Medicine, Charité-University Medicine Berlin, Center for Muscle and Bone Research, Free University Berlin, Hindenburgdamm 30, 12203 Berlin, Germany; ^5^Center for Cardiovascular Research (CCR), and Department of Experimental Medicine, Charité-University Medicine Berlin, Hessische Straße 3-4, 10115 Berlin, Germany; ^6^Institute of Molecular Cell Biology, Center for Molecular Biomedicine, Jena University Hospital, Hans-Knöll-Straße 3-4, 07745 Jena, Germany

## Abstract

Collateral growth, arteriogenesis, represents a proliferative mechanism involving endothelial cells, smooth muscle cells, and monocytes/macrophages. Here we investigated the role of Density-Enhanced Phosphatase-1 (DEP-1) in arteriogenesis *in vivo*, a protein-tyrosine-phosphatase that has controversially been discussed with regard to vascular cell biology. Wild-type C57BL/6 mice subjected to permanent left common carotid artery occlusion (CCAO) developed a significant diameter increase in distinct arteries of the *circle of Willis*, especially in the anterior cerebral artery. Analyzing the impact of loss of DEP-1 function, induction of collateralization was quantified after CCAO and hindlimb femoral artery ligation comparing wild-type and DEP-1^−/−^ mice. Both cerebral collateralization assessed by latex perfusion and peripheral vessel growth in the femoral artery determined by microsphere perfusion and micro-CT analysis were not altered in DEP-1^−/−^ compared to wild-type mice. Cerebrovascular reserve capacity, however, was significantly impaired in DEP-1^−/−^ mice. Cerebrovascular transcriptional analysis of proarteriogenic growth factors and receptors showed specifically reduced transcripts of PDGF-B. SiRNA knockdown of DEP-1 in endothelial cells *in vitro* also resulted in significant PDGF-B downregulation, providing further evidence for DEP-1 in PDGF-B gene regulation. In summary, our data support the notion of DEP-1 as positive functional regulator in vascular cerebral arteriogenesis, involving differential PDGF-B gene expression.

## 1. Introduction

Protein-tyrosine-phosphatases (PTPs) are endogenous enzymes regulating major cellular processes such as cell proliferation, cell motility, and differentiation [1]. The Density-Enhanced Phosphatase-1 (DEP-1) is a ubiquitously expressed receptor-like PTP most abundantly detected in endothelial cells and various types of hematopoietic cells [2, 3]. DEP-1 was reported to increase with cell density, indicating a role in cell-density-dependent growth inhibition [4]. Mutations and loss of heterozygosity of DEP-1 have been observed in human cancers, substantiating a role in negatively regulating cell proliferation [5–7].

Arteriogenesis refers to fluid shear stress-induced rapid proliferation of preexisting arterioles and substantial vessel diameter increase within few days [8]. This process involves a complex network of both cellular and molecular dynamics, including attraction and accumulation of circulating monocytes in the onset of arteriogenesis [9]. Production of vascular endothelial growth factor (VEGF) by recruited monocytes and other bone-marrow-derived mononuclear cells displays another critical event during arteriogenesis [10]. The VEGF2-receptor (VEGFR2) and its most potent ligand VEGF-A contribute to endothelial proliferation, migration, and vascular permeability [11]. Blockage of the VEGF receptor was reported to attenuate arteriogenesis in rats [12, 13]. Among the many relevant vascular functions induced by VEGF-A, endothelial expression of platelet-derived growth factor- (PDGF-B) has been described, impacting on vascular smooth muscle cell (VSMC) proliferation [14], which represents a general crucial component in vascular remodeling [15–18].

DEP-1 has been described as negative regulator of the PDGF-*β* receptor (PDGF-*β*R), the VEGF-receptor, and other receptor tyrosine kinases (RTKs) [15, 19–21]. Functional inhibition of DEP-1 has been shown to contribute to endothelial cell proliferation [22]. DEP-1 depletion resulted in increased VEGFR2 tyrosine phosphorylation [23]. Interestingly, this did not result in overall stimulation of VEGFR2-dependent signaling. In contrast, depletion of DEP-1 showed impairments in c-src and Akt/PKB activation/phosphorylation [24]. However, little mechanistic insight into DEP-1 function *in vivo*, in particular induced vascular remodeling processes, is available. While being a negative regulator in PDGF signaling and VSMC proliferation and chemotaxis [15, 20], conflicting data have been reported with regard to VEGF signaling and endothelial cell responses [22–24]. Thus, here we analyzed the biological function of DEP-1 *in vivo* on collateral growth using DEP-1^−/−^ mice. Cerebral arteriogenesis was assessed with a recently established permanent common carotid artery occlusion model in mice [25]. Furthermore, we subjected DEP-1^−/−^ mice and wild-type littermates to a peripheral arteriogenesis model based on occlusion of the femoral artery [26]. 

## 2. Materials and Methods

### 2.1. General Surgical Procedure and Anesthesia

Adult male DEP-1 knockout (C57BL/6 background; Deltagene, strain: Ptprj (t736)) and wild-type C57BL/6 mice (25–30 g) were used. All surgical procedures were performed under anesthesia initiated by 100 mg/kg ketamine and 10 mg/kg xylazine i.p. and maintained by ventilation with 1-2% isoflurane/~30% oxygen/~70% nitrous oxide. All experiments were in accordance with institutional guidelines and were approved by the Landesamt für Gesundheit und Soziales (LAGeSo, Berlin, Germany).

Similarly as previously described, DEP-1 knockout mice did not display any obvious phenotypic abnormalities [27, 28].

### 2.2. Cerebral Hypoperfusion Arteriogenesis Model

Cerebral hypoperfusion was induced by unilateral permanent left common carotid artery occlusion (CCAO), as previously described [25]. Cerebrovascular architecture was determined 7 days *after operation* after maximal vasodilation with 1 mg/kg adenosine using a modified postmortem latex perfusion [29]. External vessel diameters of the vessel branches of the *circle of Willis* were measured with a stereozoom microscope (Leica MZ6). Cerebrovascular hemodynamic reserve capacity (CVRC) was determined after acetazolamide (30 mg/kg Diamox, Sanofi-Aventis) induced standardized vasodilatation and continued ipsilateral laser doppler flowmetry measurements 7 days after CCAO.

### 2.3. Peripheral Arteriogenesis Model

The right femoral artery was ligated proximal and distal to the lateral caudal femoral artery as previously described [30]. Vessel diameters and collateral vessel density were determined 7 days after femoral artery occlusion (FAO) using micro-CT analysis or microsphere perfusion. Micro-CT was done by cannulating the abdominal aorta, perfusion with 1 mg/kg adenosine for maximal vasodilation, and subsequent injection of Microfil (MV-122, Flow Tech, Carver, MC, USA). After PET/CT (20 *μ*m resolution, Inveon, Siemens) the arterial collateral network was visualized with Amira 5.5.5 software (Visage Imaging, Berlin, Germany). Vessel diameter measurements were done on the basis of three-dimensional (3D) images. Microsphere perfusion was analyzed after delivery of 0.03 mg/mL/min adenosine via the aortic bifurcation for maximal vasodilation of the hindlimb arterial network; perfusing with fluorochrome-labeled microspheres was done at different compression intensities. After isolation and enzymatic digestion of the hindlimb muscle tissue, fluorescent microspheres were isolated and subsequently counted by FACS analysis. The counts were normalized towards a determined number of additionally added blue microspheres.

### 2.4. Gene Expression Analysis

Ipsilateral anterior cerebral arteries (ACAs) were isolated and lysed in RLT buffer (Qiagen). Total RNA extraction was performed with the RNAeasy Mini Kit (Qiagen). Random primers and Super Script II (Invitrogen) were used for cDNA synthesis. Gene expression analysis by quantitative real-time polymerase chain reaction (qPCR) (SybrGreen) was done in duplicate or triplicate with an Mx3000P cycler (Stratagene; Agilent Technologies, La Jolla, CA,USA) and normalized to 18S. Primer sequences (all used at final concentration of 100 nM) were as follows: PDGF-*β*R: CCTGCAGAGACCTCAAAAGTAGGT, GCTCTCCTCCTTGGAACTATTCC; PDGF-B: AATAACCGCAATGTGCAATGC, TCGCACAATCTCAATCTTTCTCA; PDGF-D: ATCCGGACACTTTTGCGACT, CATGGCCATTGCTTGTCACC; VEGFR2: TTGCCTGGTCAAACAGCTCA, ATGGTCTCGCCAATGGTTGT; VEGF-A: ACTTGTGTTGGGAGGAGGATGTC, AATGGGTTTGTCGTGTTTCTGG; PTP1B: CGGGAGGTCAGGGACCTT, GGGTCTTTCCTCTTGTCCATCA; TC-PTP: ACCTGCAGTGATCCATTGCA, ATCAGAACAAGACAGGTATCTACAAGAGA; DEP-1: GCAGTGTTTGGATGTATCTTTGGT, CTTCATTATTCTTGGCATCTGTCCTT; SHP-2: CCTCAACACAACTCGTATCAATGC, TGTTGCTGGAG-CGTCTCAAA; FGF2: GCCAACCGGTACCTTGCTAT, GTCCAGGTCCCGTTTTGGAT; TGF*β*1: CTGCTGACCCCCACTGATAC, GTGAGCGCTGAATCGAAAGC (forward primer, reverse primer, resp.). 

### 2.5. Cell Culture and siRNA

Endothelial cells (CRL-2181) were obtained from ATCC, and cells were cultured as suggested by the supplier. DEP-1 knockdown was done by delivery of DEP-1 and nontargeting siRNA (both 10 *μ*M; Dharmacon) using RNAiMAX (Invitrogen). Representative microphotographs (Keyence BZ-9000, magnification: 20x) were taken 48 hours after transfection in order to determine endothelial cell density and morphology.

### 2.6. Immunoblotting

Immunodetection of DEP-1 from spleen tissue or endothelial cells was performed after Lectin-WGA-Sepharose (Sigma) precipitation and subsequent immunoblotting using standard procedures. Anti-CD148 (R&D Systems), anti-alpha-tubulin (Sigma), or anti-beta-actin (Santa Cruz) antibodies were used.

### 2.7. Phosphatase Assay

Liver (as marker tissue) was lysed in a dounce homogenizer using lysis buffer adjusted to tissue weights (150 mM NaCl, 25 mM C_2_H_3_NaO_2_, 1% NP-40, 10 mM DTT, and aprotinin (4 *μ*g/mL)). DEP-1 was immunoprecipitated with anti-CD148 (1 *μ*g, R&D systems) in an end-over-end reaction at 4°C overnight. Immunoprecipitates were collected by Dynabeads Protein G (Invitrogen) for 1 hour. After washing twice with lysis buffer, and once with reaction buffer the precipitates were resuspended in 50 *μ*L reaction buffer, and DTT was added to a final concentration of 50 mM. PTP activity was determined using a ^32^P-labeled src-optimal peptide as substrate. Measurements were performed in duplicate, and the amount of ^32^P-labeled radioactivity being released from the peptide after 7 min of incubation at 30°C was measured.

### 2.8. Statistical Analysis

Data are expressed as mean ± standard deviation or standard error of the mean. *P* values were calculated using unpaired Student's *t*-test. *P* < 0.05 was considered as statistically significant.

## 3. Results

### 3.1. Permanent CCAO Induces Arteriogenesis in Mice

**Figure 1:**
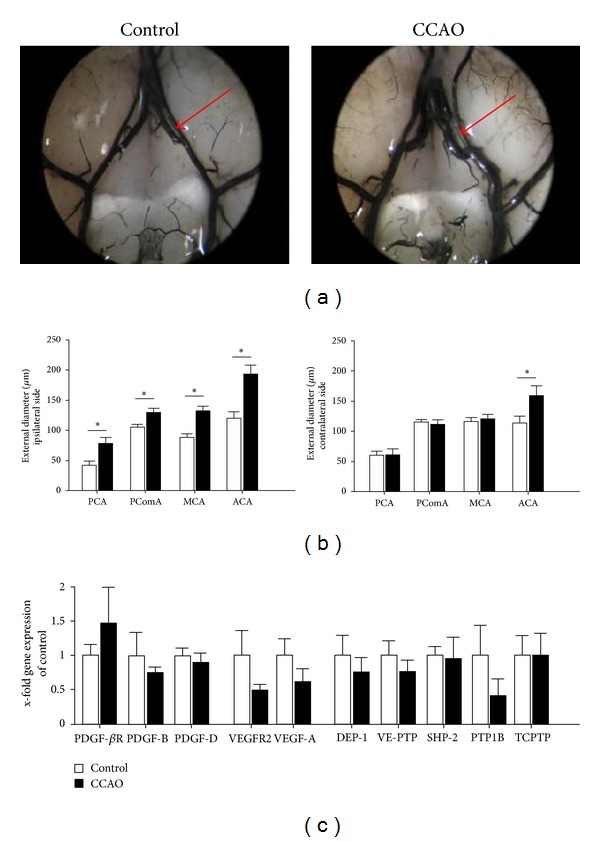
(a) Control mice (*n* = 5) and mice after CCAO (*n* = 5) were subjected to latex perfusion for visualization of the collateral vessel network. Red arrows indicate the ipsilateral ACA. (b) Quantification of the external vessel diameters (*P < 0.05 versus control mice). (c) Gene expression analyses of the PDGF-*β*R, VEGFR2, PDGF- and VEGF-ligands, and receptor-targeting PTPs in the ACA (*n* = 4). PCA: posterior cerebral artery, PComA: posterior communicating artery, MCA: middle cerebral artery, and ACA: anterior cerebral artery.

CCAO was followed by significant induction of collateral growth in the ipsilateral posterior cerebral artery (PCA), posterior communicating artery (PComA), the middle cerebral artery (MCA), and the anterior cerebral artery (ACA) of the *circle of Willis* (Figures 1(a) and 1(b)). The ACA was the only artery with significant growth at both the contralateral and the ipsilateral hemispheres, with largest growth induction in general.

Consequently, further gene expression analyses were done in ACA tissues regarding genes implicated in vessel remodeling processes. Based on the impact of the PDGF and VEGF signaling for vascular remodeling [10, 15, 31], we performed gene expression profiling of the PDGF-*β*R and its cognate ligands, PDGF-B, PDGF-D, as well as VEGFR2 and VEGF-A in the ACA. By comparing CCAO and control animals a slight increase in PDGF-*β*R transcripts in the ipsilateral ACA was detected (Figure 1(c)). No changes were visible regarding PDGF-B and PDGF-D, while VEGFR2 and VEGF-A were reduced in the ipsilateral ACA. Additionally, we examined the expression of PTPs targeting PDGF-*β*R and VEGFR2. PTP1B transcripts were decreased, suggesting a reduction of this phosphatase which is known as negatively regulating cell growth. DEP-1, TC-PTP, SHP-2, and VE-PTP remained similar between control and CCAO animals (Figure 1(c)). 

Taken together, no statistically significant differential gene expression of the PDGF-*β*R, the VEGFR2, its cognate ligands PDGF-B, PDGF-D, and VEGF-A, respectively, and targeting PTPs was detected in the ACA following CCAO, despite valid proarteriogenic effects in the cerebral vasculature. We further examined the impact of loss of DEP-1 in arteriogenesis.

### 3.2. Analyses of Cerebral Arteriogenesis in DEP-1^−/−^ versus Wild-Type Mice

**Figure 2:**
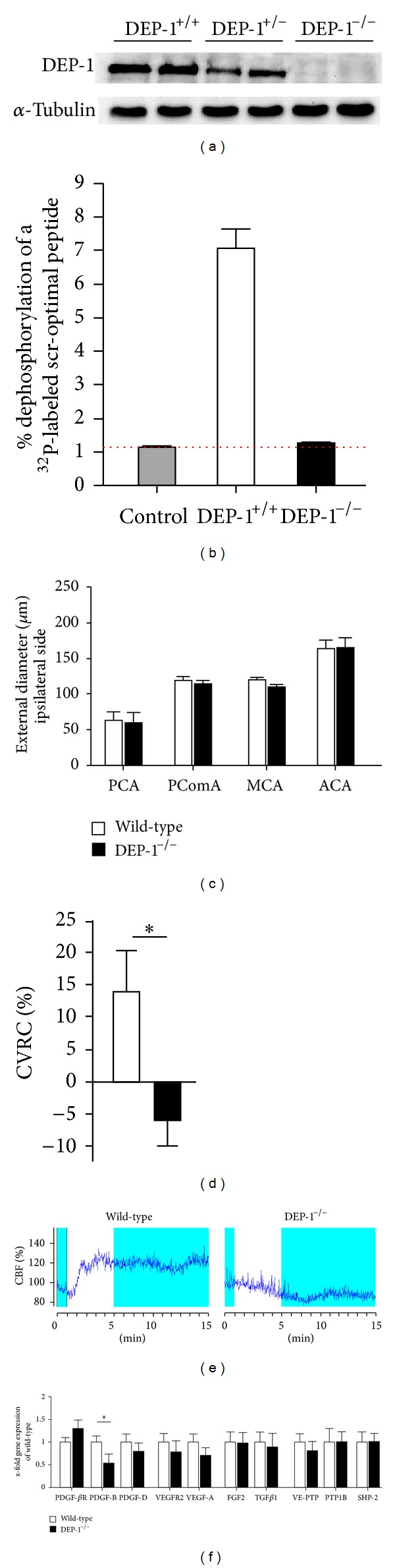
(a) Spleen lysates of DEP-1^+/+^, heterozygous, and DEP-1^−/−^ mice were precipitated; immunoblots with anti-DEP-1 antibodies were done. Equivalent protein amounts were immunoblotted against *α*-tubulin as loading control. (b) DEP-1 was immunoprecipitated and processed to a dephosphorylation assay of a radioactive labeled src-optimal peptide. A species-matched unspecific IgG served as Control. Shown is one representative experiment of % dephosphorylation in tissues derived from DEP-1^+/+^ and DEP-1^−/−^ mice (*n* = 3). (c) Quantification of the external vessel diameters (*n* = 7–10). (d) Cerebrovascular reserve capacity (CVRC) determined by laser doppler flowmetry measurement 7 days after CCAO surgery (*n* = 10) (*P < 0.05 wild-type versus DEP-1^−/−^ mice). (e) Representative measurements of the cerebrovascular blood flow (CBF) dynamics in wild-type and DEP-1^−/−^ mice. Initial 60 seconds was defined as baseline and minutes 5–15 was calculated as relative CBF alteration. (f) Gene expression analyses in the ipsilateral ACA of wild-type and DEP-1^−/−^ mice by qPCR (*n* = 9) (*P < 0.05 wild-type versus DEP-1^−/−^ mice).

Missing expression of DEP-1 protein in DEP-1^−/−^ mice was confirmed by immunoblotting analyses of spleen extracts (Figure 2(a)). In addition, DEP-1 dephosphorylating ability of a tyrosine-phosphorylated src-optimal peptide was lost in DEP-1^−/−^ mice (Figure 2(b)).

Analogous to our previous experiments, wild-type and DEP-1^−/−^ mice underwent CCAO surgery. Among the previously examined cerebral vessels (MCA, PCA, PComA, and ACA) no morphological differences between wild-type and knockout mice were observed (Figure 2(c)). In addition to the assessment of vessel architecture, we analyzed blood flow by cerebrovascular hemodynamic reserve capacity (CVRC) for functional analyses 7 days after CCAO. CVRC was significantly impaired in DEP-1^−/−^ mice, suggesting a positive regulatory impact of DEP-1 (Figure 2(d)).  Figure 2(e) shows representative flow patterns for up to 15 min following acetazolamide injection to induce maximal vasodilatation in wild-type and DEP-1^−/−^ mice, clearly demonstrating loss of CVRC in knockout mice.

**Figure 3:**
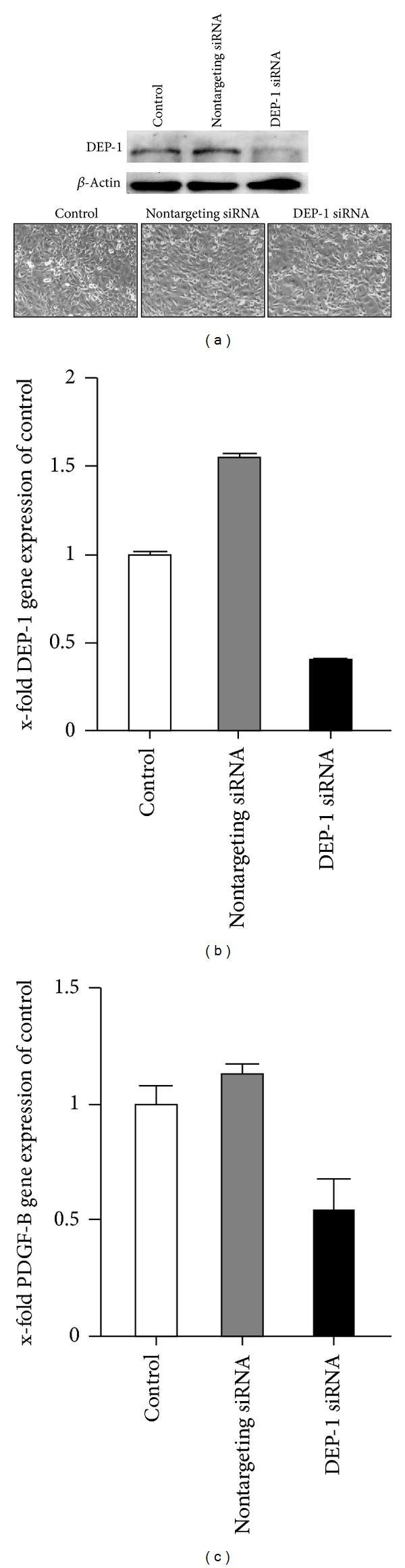
(a) Endothelial cells were transfected with DEP-1 siRNA or nontargeting siRNA for 48 hours. Representative microphotographs were taken 48 hours after transfection. Protein lysates were WGA precipitated and immunoblotted against DEP-1. Beta-actin immunoblots served as loading control. Representative qPCR analyses of DEP-1 (b) and PDGF-B (c) expressions in endothelial cells 48 hours after DEP-1 siRNA knockdown are depicted (*n* = 3).

Gene expression analyses of the PDGF-*β*R, PDGF-D, VEGFR2, and VEGF-A showed no difference between wild-type and DEP-1^−/−^ mice in the ipsilateral ACA (Figure 2(f)). In addition, transcript levels of fibroblast growth factor 2 (FGF2) and transforming growth factor beta1 (TGF*β*1), which both have been implicated in arteriogenesis, were not significantly different in DEP-1 knockout compared to wild-type mice [32, 33]. Furthermore, RTK-antagonizing PTP1B, SHP-2, and VE-PTP remained equal, excluding a compensatory differential PTP expression due to DEP-1 knockout. However, PDGF-B transcripts were significantly reduced in DEP-1^−/−^ mice, suggesting an impact of DEP-1 on PDGF-B gene expression, resulting in altered vasodilatatory capacity. Since PDGF-B expression in the vascular wall is, at least in part, driven by the endothelium, we analyzed whether knockdown of DEP-1 in endothelial cells indeed leads to reduced gene expression. As shown in Figures 3(a) and 3(b), transfection of siRNA against DEP-1 in mouse endothelial cells was effective and resulted in significantly lower DEP-1 expression, without explicit impact on cell density and/or morphology after 48 hours. This was accompanied by reduced PDGF-B gene expression (Figure 3(c)), substantiating our *in vivo* findings. 

Together, we detected impaired CVRC in DEP-1^−/−^ mice, along with reduction of PDGF-B gene expression, while no morphological changes in vessel architecture were visible. Next we assessed whether the negative impact of DEP-1 knockout was restricted to cerebral arteriogenesis. Thus, the role of DEP-1 was assessed in peripheral collateral growth.

### 3.3. Analyses of Peripheral Arteriogenesis in DEP-1^−/−^ versus Wild-Type Mice

Peripheral collateral growth was investigated after FAO leaving the left femoral artery untouched as an internal control. Quantification of peripheral collateral growth was determined 7 days after FAO using microsphere perfusion and micro-CT imaging.

**Figure 4:**
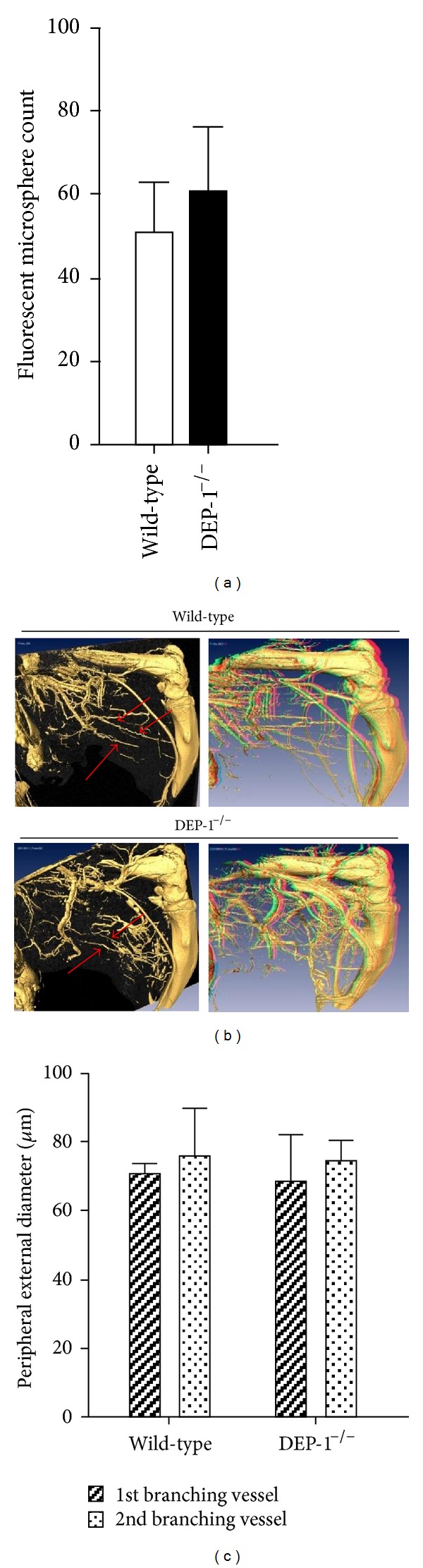
(a) Collateral perfusion index after FAO. Wild-type and DEP-1^−/−^ animals were perfused with fluorescent microspheres at different compression intensities after maximal vasodilatation. Limb muscles were lysed, and microsphere counts (FACS) were normalized to a defined number of blue spheres as outlined in Materials and Methods (wild-type *n* = 5, knockout *n* = 4). (b) Representative micro-CT-based images of the femoral artery and interconnecting peripheral hindlimb vessels (red arrows, left) as well as 3D images for visualization of vascular intratissue characteristics (right). (c) Quantifications of external peripheral vessel diameter measurements are shown.

Microfil perfusion analysis revealed no difference between DEP-1^−/−^ and wild-type mice (Figure 4(a)). Further, visualization of the peripheral collateral network in the mice hindlimb by micro-CT imaging and subsequent generation of a 3D model of the femoral artery and branching vessels were performed. As depicted in Figure 4(b), showing representative 2D and 3D mirrored images of the right hindlimb, no differences were detectable between groups. In addition, quantification of digital 3D pictures is shown in Figure 4(c), substantiating that functional disruption of the DEP-1 gene did not translate into changes in induced peripheral collateral growth.

Thus, in contrast to cerebral arteriogenesis, we could not detect a major role of DEP-1 in peripheral collateral growth.

## 4. Discussion

Arteriogenesis, collateral growth, has attracted significant interest as a potential therapeutic option to circumvent circulatory deficits. It involves several cell types, together impacting on vascular growth. In our study we hypothesized that DEP-1, a protein-tyrosine-phosphatase (PTP) primarily negatively regulating a variety of receptor tyrosine kinases, may play a role in experimentally induced cerebral and peripheral arteriogenesis. Here we demonstrate that loss of DEP-1 impairs cerebral arteriogenesis along with reduced PDGF-B expression, while peripheral collateral growth is not affected. 

We assessed DEP-1 deficiency in a mouse model [25] capable of inducing cerebral arteriogenesis by CCAO. In order to be able to detect either a positive or a negative impact on collateral growth due to loss of DEP-1, day 7 after operation was chosen as a read-out for collateral growth. Arteriogenesis was detectable in all analyzed vessels branching off the *circle of Willis*. In contrast to a three-vessel occlusion model in rats [34, 35], the ipsilateral ACA but not the PCA diameter was characterized by the highest diameter increase after 7 days. This difference might be partially based on different flow dynamics due to the differences in surgery. However, the overall increase in vessel diameter confirmed successfully induced cerebral arteriogenesis in mice. 

Expression of proteins of the PDGF signaling pathway including the PDGF-*β*R, ligands, and the PDGF-receptor-antagonizing PTPs PTP1B, DEP-1, TC-PTP, and SHP-2 was investigated. The importance of PDGF-*β*R mediated signaling *per se* in VSMC proliferation and vessel development/remodeling has been impressively emphasized in PDGF-*β*R null mice [36, 37]. We detected only a very mild if any regulation of the PDGF-*β*R and its ligands or the receptor-antagonizing PTPs SHP-2, TC-PTP, PTP1B, and DEP-1 7 days following CCAO in the ACA. The tendency of a reduced PTP1B expression might implicate and confirm its role as negative regulator in vascular proliferation and remodeling-associated processes as described in neointima formation [38]. Even though widely expressed, immunohistochemistry analyses have revealed that DEP-1 is highly expressed in endothelial cells, where it interferes with VEGF-receptor signaling, thereby contributing to VEGF-dependent expression of PDGFs [2, 14, 22]. However, gene expression profiling in our study did not show VEGF ligand and receptor expression changes in the ACA induced by CCAO. However, we cannot rule out that time points early after induction of collateral growth would unravel involvement of genes including the VEGF and PDGF families. Additionally, other factors/genes not being analyzed in our study might also be of relevance, accounting for the observed vessel growth in particular with regard to the ACA. 

Dynamic changes of PTPs have been described in animal models of vascular remodeling including restenosis and pulmonary hypertension [15, 31]. With 38 classical PTPs being identified in the human genome, which all share a catalytic signature motif V/I H C S X G [1], functional redundancy of DEP-1 in tissue remodeling processes might exist. Indeed, functional redundancy in which loss of one receptor-like PTP is compensated by another PTP has been provided earlier [39]. However, we excluded counterregulatory differences in VE-PTP, SHP-2, and PTP1B expressions. As DEP-1, these PTPs have all been described in the vascular wall as well as binding to PDGF and VEGF receptors.

Important targets in vascular remodeling and cell proliferation negatively regulated by DEP-1 such as the VEGF, PDGF, and EGF receptors, as well as extracellular signal-regulated kinases, have been highlighted [15, 20, 21, 31]. We therefore hypothesized an impact on arteriogenesis in mice deficient for DEP-1. Applying two different arteriogenesis models in mice with both functional and morphological read-outs, a major role of DEP-1 in collateral vessel growth could not be detected in the periphery, while cerebral arteriogenesis was functionally impaired. This impairment was accompanied by significantly reduced PDGF-B gene expression, while other growth factors (VEGF-A, PDGF-D, FGF2, and TGF*β*1) were not affected. Indeed, homodimerized PDGF-B, PDGF-BB, has been implicated in regulating the vascular tonus. Reduction of PDGF-BB was effective in preventing vasospasms, and direct vasoconstrictive effects on cerebral arteries were shown in rabbits [40]. In our study, a direct link between DEP-1 and PDGF-B gene expressions was implicated by siRNA-DEP-1 knockdown in endothelial cells, significantly reducing PDGF-B transcripts. In contrast to CVRC, DEP-1^−/−^ mice showed no significant changes either in cerebral vessel morphology and gene expression or in peripheral collateral architecture and microsphere perfusion after hindlimb occlusion. This difference suggests organ-specificity possibly linked to different flow patterns, differential (basal) gene expression, and/or regulated diverse involvement of cellular components. Furthermore, endothelial cell phenotypes significantly differ, depending on the origin of the vascular bed [41]. Thus, these factors might cause differences in susceptibility to defined (vascular) pathological and/or inducible conditions.

Vascular remodeling processes are multicellular events with a highly organized time course and numerous signaling pathways involved. Several PTPs like PTP1B and SHP-2 have been implicated in positively regulating PDGF-*β*R-dependent c-src and VEGFR2 mediated Akt and c-src activation and receptor-independent activation of Ras [26, 42, 43]. Indeed, Oshikawa et al. demonstrated siRNA against DEP-1 resulting in enhanced VEGF-induced VEGFR2 tyrosine phosphorylation [44]. However, recent data demonstrated that knockdown of DEP-1 impaired c-src-dependent cellular responses and Akt phosphorylation induced by VEGF [23, 24]. Depletion of DEP-1 and potential positive effects on collateral vessel growth by removing the negative regulatory function of DEP-1 might be at least partially counteracted by the simultaneous loss of positive DEP-1 signaling [24], leading to unchanged vessel architecture, while in contrast DEP-1 deficiency clearly results in functional cerebral vasoregulatory changes. 

## 5. Conclusions

Taken together, our study demonstrates that knockout of the protein-tyrosine-phosphatase DEP-1 in mice is followed by reduced anterior cerebral artery PDGF-B gene expression and impaired cerebrovascular reserve capacity. Thus, this suggests that DEP-1 is acting as a positive functional regulator in cerebral arteriogenesis.
